# Supplementary material for: Glutamine Availability Controls BCR/Abl Protein Expression and Functional Phenotype of Chronic Myeloid Leukemia Cells Endowed with Stem/Progenitor Cell Potential
Source: Cancers (Basel). 2021 Aug 30;13(17):4372. doi: 10.3390/cancers13174372 (PMC8430815; doi:10.3390/cancers13174372)
Supplement: Supplementary file 1 [file cancers-13-04372-s001.zip › cancers-1321193-supplementary.pdf]

cancers

MDPI

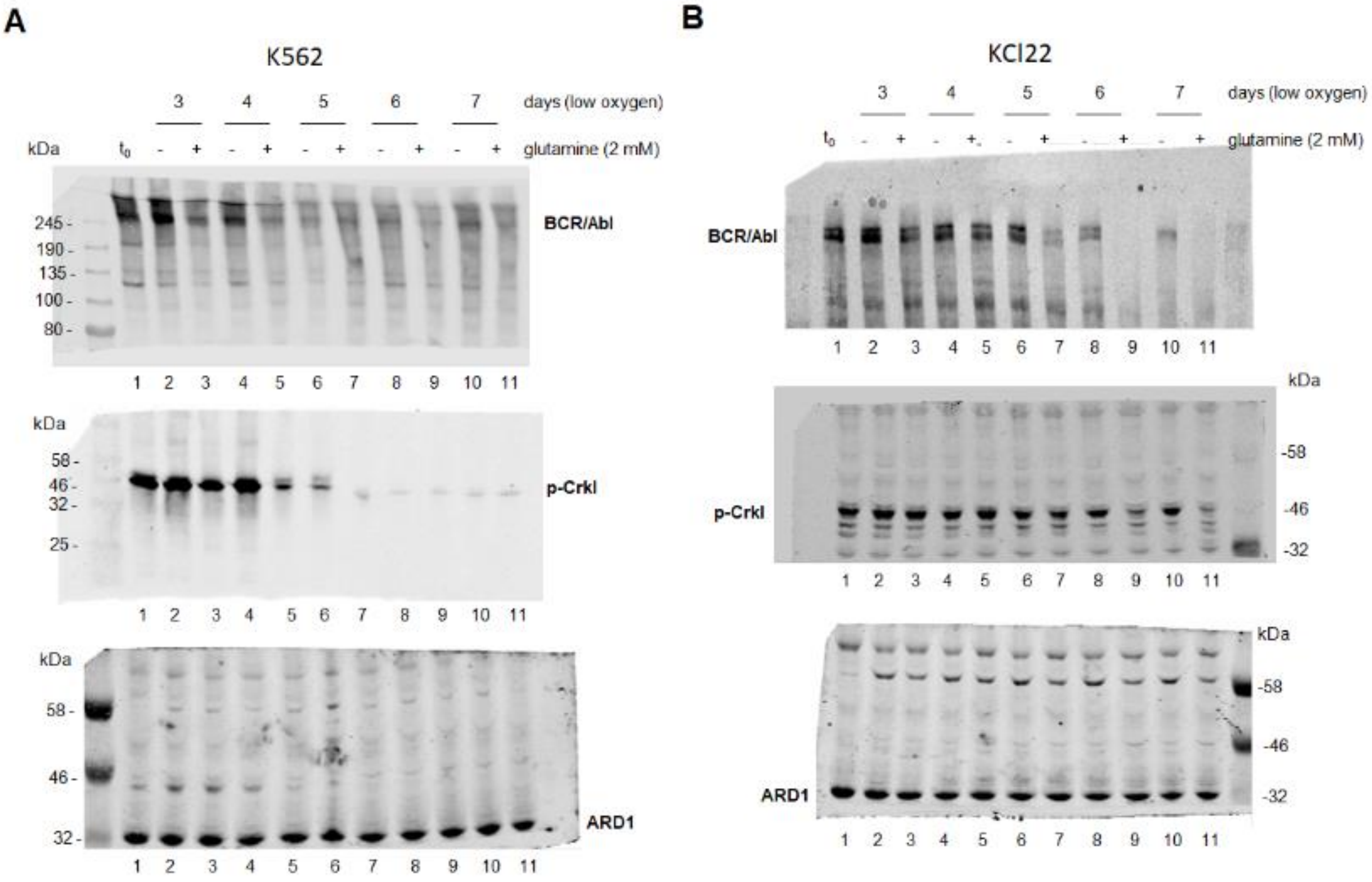

C

K562	BCR/Abl	p-Crkl	ARD1
1	86	113	93
2	119	127	93
3	58	85	95
4	91	117	89
5	56	42	94
6	43	33	91
7	58,7	8	94
8	62	4,8	91
9	40	4,5	84
10	70	4,9	85
11	47	6,4	77

D

K562	BCR/Abl	p-Crkl
1	100	100
2	138	109
3	66	82
4	110	113
5	64	49
6	51	40
7	67	15
8	74	12
9	51	14
10	89	13
11	66	17

E

KCL22	BCR/Abl	p-Crkl	ARD1
1	177	113	136
2	201	128	128
3	157	115	122
4	175	101	126
5	162	114	137
6	164	94	136
7	70	90	133
8	93	102	133
9	9	52	130
10	56	86	112
11	21	42	120

F

KCL22	BCR/Abl	p-Crkl
1	100	100
2	120	120
3	98	113
4	107	95
5	90	100
6	92	84
7	40	80
8	54	90
9	5,4	48
10	40	91
11	13,8	42

Figure S1. Original blots relative to Figure 2G (**A**) and Figure 2H (**B**). Each Western blot band was quantified using densitometric analysis (**C, E**) and the intensity ratio with respect to the corresponding t0 band was calculated after normalization for the ARD1 value (**D, F**).

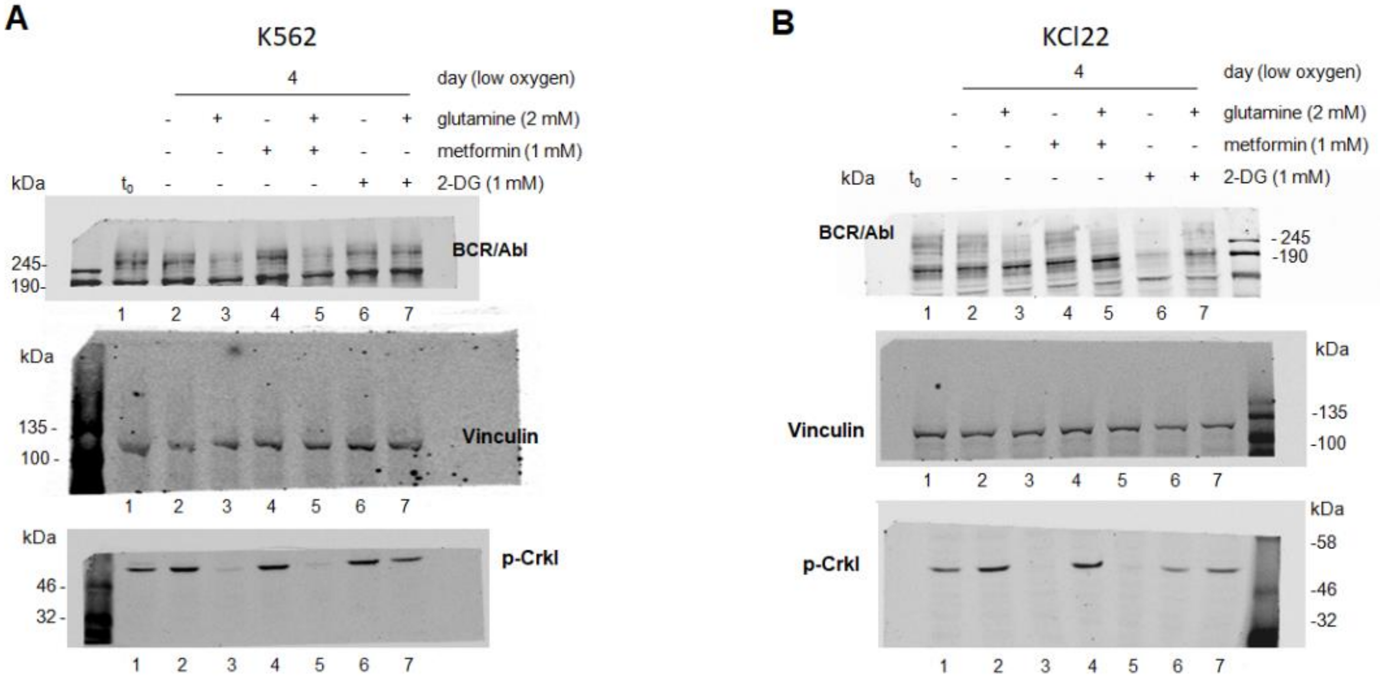

C

K562	BCR/Abl	P-Crkl	Vinculin
1	121	47	90
2	92	65	56
3	51	12	57
4	121	70	76
5	55	18	71
6	96	74	85
7	105	46	86

D

K562	BCR/Abl	P-Crkl
1	100	100
2	122	223
3	66	40
4	118	176
5	57	48
6	84	167
7	91	101

E

KCL22	BCR/Abl	P-Crkl	Vinculin
1	112	30	87
2	98	46	77
3	56	6,7	78
4	121	46	91
5	67	7,9	84
6	46	17	67
7	71	32	79

F

KCL22	BCR/Abl	P-Crkl
1	100	100
2	100	176
3	55	26
4	103	147
5	62	28
6	53	73
7	70	117

Figure S2. Original blots relative to Figure 4C (**A**) and Figure 4D (**B**). Each Western blot band was quantified using densitometric analysis (**C, E**) and the intensity ratio with respect to the corresponding t0 band was calculated after normalization for the Vinculin value (**D, F**).

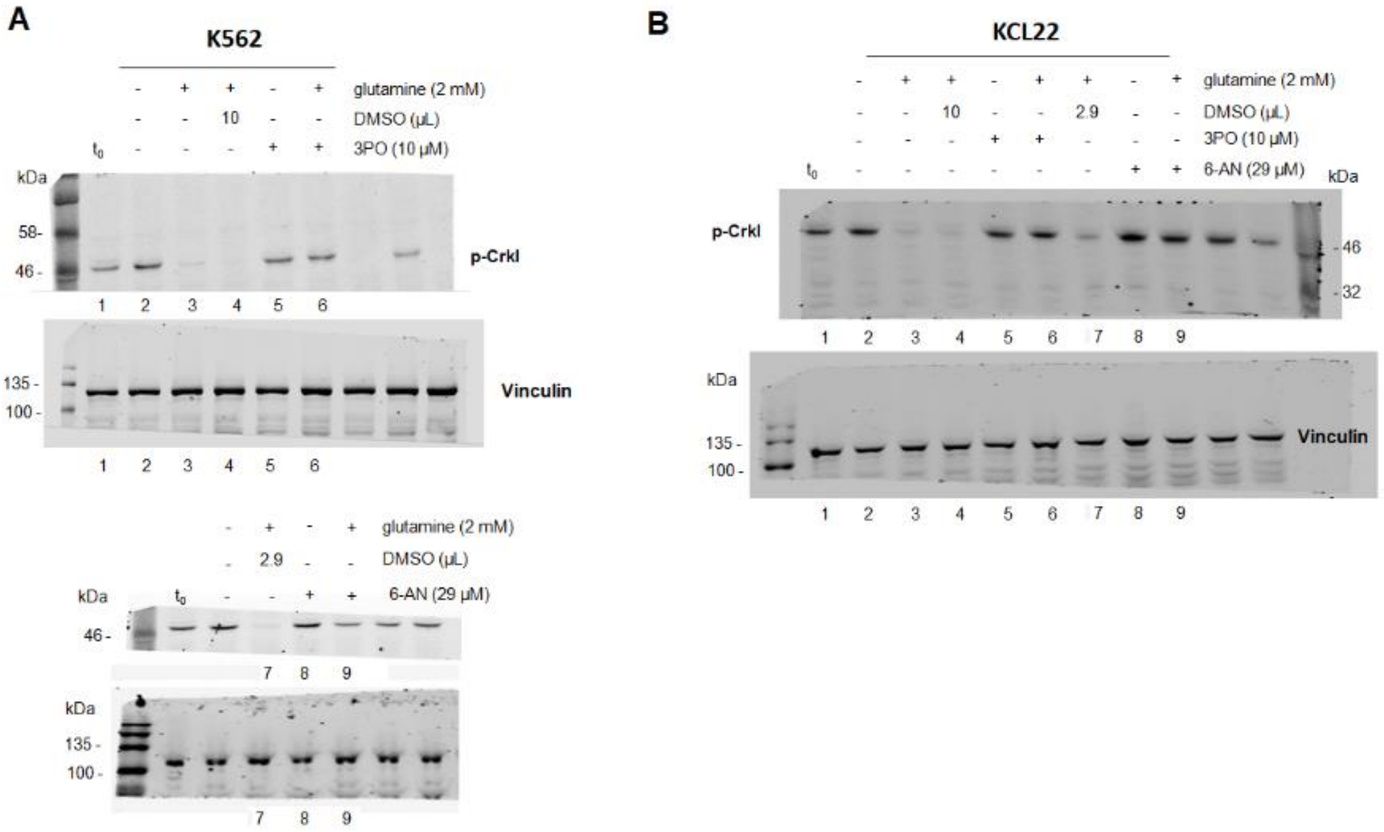

C

K562	P-crkl	Vinculin
1	27	42,4
2	34,8	42,7
3	9,5	52,2
4	8	53,8
5	27,9	47,5
6	27	55,7
7	9,3	46,2
8	35,4	38,5
9	16,6	44,1

D

K562	P-crkl
1	100
2	127
3	28,4
4	23,2
5	92
6	76
7	31
8	144
9	60

E

KCl22	P-crkl	Vinculin
1	105	113
2	123	81
3	42	84
4	45	88
5	123	91
6	126	103
7	57	91
8	158	112
9	132	97

F

KCL22	P-crkl
1	100
2	162
3	54
4	55
5	145
6	131
7	66
8	151
9	146

Figure S3. Original blots relative to Figure 5A (**A**) and Figure 5B (**B**). Each Western blot band was quantified using densitometric analysis (**C, E**) and the intensity ratio with respect to the corresponding t0 band was calculated after normalization for the Vinculin value (**D, F**).

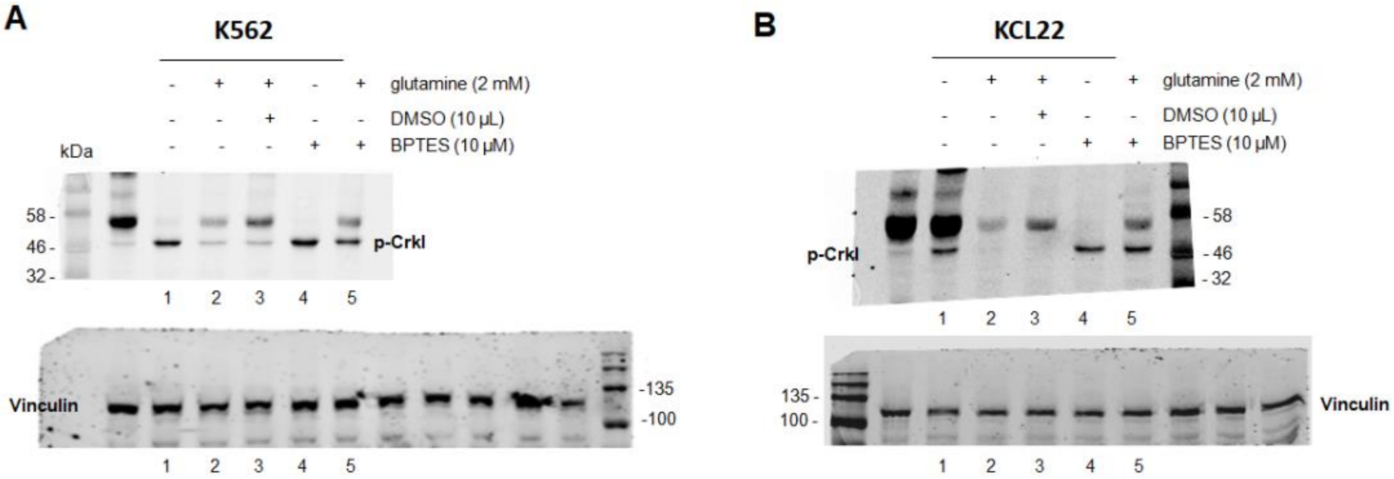

C

K562	p-crkl	Vinculin
1	161	232
2	43	202
3	56	195
4	197	198
5	145	194

D

K562	p-crkl
1	100
2	30,7
3	41,3
4	143
5	107

E

KCl22	p-crkl	Vinculin
1	186	151
2	35	151
3	49	163
4	176	159
5	188	178

F

KCL22	p-crkl
1	100
2	18,6
3	24,4
4	89
5	86

Figure S4. Original blots relative to Figure 7A (**A**) and Figure 7B (**B**). Each Western blot band was quantified using densitometric analysis (**C, E**) and the intensity ratio with respect to untreated cells (no glutamine, no DMSO, no BPTES) was calculated after normalization for the Vinculin value (**D, F**).
